# An Address on Aseptic Midwifery

**Published:** 1899-09-16

**Authors:** Robert Jardine

**Affiliations:** Physician to the Hospital


### Hospital Clinics and Medical Progress.

AN ADDRESS ON ASEPTIC MIDWIFERY,
Delivered in the Glasgow Maternity Hospital, by Robert Jardine, M.D., &c., Physician to the Hospital.

Last session I began the work by giving an address on "Aseptic Midwifery," and I do not think I can do better on this occasion than to give you a few of the reasons why I consider aseptic methods preferable to others in obstetric work.

Aseptic midwifery is not a new departure at all. As a matter of fact, it was first practised over fifty years ago by Semmelweis, to whom we owe so much. He was the first to recognise that puerperal fever was nothing more nor less than blood poisoning in a puerperal woman, and that the poison gained entrance through the genital tract. As assistant in the First Midwifery Clinique in Vienna, where the mortality was very high, he had ample opportunities of studying puerperal sepsis. The women in the First Clinique were attended by medical students, who were at the same time working in the dissecting-rooms. In the Second Clinique midwives attended the cases. The mortality in the First Clinique was much greater than in the Second. While searching for an explanation of this, light was suddenly thrown upon the subject in a very tragic manner. The Professor of Forensic Medicine pricked his finger at a post-mortem examination, and died of blood poisoning, as many a man had done before and has done since. Semmelweis recognised the identity of the two diseases, and at once the explanation of the higher mortality among the students' cases was plain to him. The students were conveying infection from the post-mortem and dissecting rooms to their patients. The source of contamination having been recognised, the next step was to guard against it. He did this by making the students wash their hands carefully in chlorine solutions; he afterwards substituted solutions of chlorinated lime. In a short time the mortality among the students' cases was reduced far below that among the midwives, proving the soundness of his views.

One would imagine that these views would have at once been accepted by all obstetricians. By some they were, but by many of the leaders of the profession they were either ignored or actively opposed, with the result that poor Semmelweis had to leave Vienna. He afterwards became Professor of Midwifery in the University of Pesth. In 1861 he published a work entitled "The Etiology, Nature, and Prophylaxis of Puerperal Fever." This work was coldly received, and he became so embittered by the treatment meted out to him and his works that he entered into a violent controversy with several leaders of the profession. In the end his mind gave way, and he was confined in an asylum, where he shortly afterwards died. By the irony of fate his death was due to blood-poisoning. He died in 1865, but he had not lived in vain. In 1894 a monument, subscribed to from all parts of the world, was raised to his memory. He met with the fate of many reformers, but his name and fame will live for ever, while the names of those who so bitterly opposed him are already forgotton.

To Semmelweis bacteriology was, of course, an unknown science, yet his views as to the origin of puerperal fever were correct, and his method of prevention by strict cleanliness is the one that holds the field to-day. Had his views been adopted at the time, the era of antiseptics would have dawned ere it did. The year 1847 was a notable one in obstetrics. It saw the introduction of chloroform by Sir J. J. Simpson, and the first intelligent use of antiseptics by Semmelweis.

You are all perfectly familiar with the *rôle* antiseptics play in the domain of surgery. By the means of antiseptics the dangers of surgical operations have been very much lessened, so that many operations which before the days of antiseptics could not be attempted with much hope of success are now performed daily with little risk to the patients. I need only instance operations on one region of the body, viz., the peritoneal cavity, which in my student days were only attempted in fear and trembling, especially by ordinary surgeons. At present I am afraid the peritoneum is treated, perhaps, with just a little too much contempt as a portion of the body which will stand any amount of handling. Of late years we do not hear so much about antiseptic surgery, as aseptic surgery has become the fashion. While antiseptic and aseptic methods have done so much for surgery, how much have they done for midwifery? In our hospital practice the success has been great, but one cannot say the same in regard to private work. In private work there has undoubtedly been an improvement from the pre-antiseptic days, but puerperal sepsis is still far too common, as is shown by the Registrar-General's reports, and also by the very large number of gynæcological cases arising from septic absorption during the puerperium. The mortality is still too high, and the morbidity markedly so. Many die, but many are left maimed for life. To the general eye the morbidity is scarcely apparent, as there is usually no external manifestation, but the patient herself unfortunately suffers all the more from the source being an internal one. Puerperal septicæmia is now recognised as a preventable disease, and we obstetricians have to face that fact, and endeavour in every way to prevent it. If we do not, we are not fulfilling our duties, and the responsibility lies with us. In the olden times the cause of this terrible disease was unrecognised, and the men of those days were groping in the dark. It is otherwise with us. We know that puerperal fever is nothing more nor less than septicæmia or blood poisoning in a puerperal woman, and that it is caused by the introduction of certain organisms into the system through some part of the genital tract. I believe that in practically all cases these organisms are introduced at the time of labour or during the puerperium. The internal origin of puerperal septicæmia, except in the case of an abscess present at the time of labour, is a myth. This is now pretty generally recognised. The aphorism that "knowledge is power" is a very true one, but knowledge brings with it grave responsibilities, responsibilities which we cannot shirk. We, who are "heirs of all the ages" in medicine and surgery, have much graver responsibilities to bear than our predecessors. They did not know what they did; we do, and the sooner we recognise this the better for our patients and for ourselves.

(*To be continued.*)

